# Protocol for regional implementation of collaborative self-management services to promote physical activity

**DOI:** 10.1186/s12913-018-3363-8

**Published:** 2018-07-17

**Authors:** Anael Barberan-Garcia, Elena Gimeno-Santos, Isabel Blanco, Isaac Cano, Graciela Martínez-Pallí, Felip Burgos, Felip Miralles, Miquel Coca, Serafín Murillo, María Sanz, Alexander Steblin, Marta Ubré, Jaume Benavent, Josep Vidal, Marta Sitges, Josep Roca

**Affiliations:** 1 Respiratory Medicine Department, Hospital Clínic de Barcelona, CIBERES, Barcelona, Catalonia Spain; 2 Institut d’Investigacions Biomèdiques August Pi i Sunyer (IDIBAPS), Universitat de Barcelona, Barcelona, Catalonia Spain; 3 Anesthesiology Department, Hospital Clínic de Barcelona, Barcelona, Catalonia Spain; 4 EURECAT – Technological Center of Catalonia, Barcelona, Catalonia Spain; 5 Endocrinology and Nutrition Department, Hospital Clínic de Barcelona, IDIBAPS, University of Barcelona, Barcelona, Catalonia Spain; 6 Cardiology Department, Hospital Clínic de Barcelona, IDIBAPS, University of Barcelona, Barcelona, Catalonia Spain; 7 Consorci d’Atenció Primària de Salut Barcelona Esquerra (CAPSBE), Barcelona, Catalonia Spain

**Keywords:** Chronic disorders, eHealth, Integrated care, Physical activity, Cardiopulmonary rehabilitation, Service adoption

## Abstract

**Background:**

Chronic diseases are generating a major health and societal burden worldwide. Healthy lifestyles, including physical activity (PA), have proven efficacy in the prevention and treatment of many chronic conditions. But, so far, national PA surveillance systems, as well as strategies for promotion of PA, have shown low impact. We hypothesize that personalized modular PA services, aligned with healthcare, addressing the needs of a broad spectrum of individual profiles may show cost-effectiveness and sustainability.

**Methods:**

The current manuscript describes the protocol for regional implementation of collaborative self-management services to promote PA in Catalonia (7.5 M habitants) during the period 2017–2019. The protocols of three implementation studies encompassing a broad spectrum of individual needs are reported. They have a quasi-experimental design. That is, a non-randomized intervention group is compared to a control group (usual care) using propensity score methods wherein age, gender and population-based health risk assessment are main matching variables. The principal innovations of the PA program are: i) Implementation of well-structured modular interventions promoting PA; ii) Information and communication technologies (ICT) to facilitate patient accessibility, support collaborative management of individual care plans and reduce costs; and iii) Assessment strategies based on the Triple Aim approach during and beyond the program deployment.

**Discussion:**

The manuscript reports a precise roadmap for large scale deployment of community-based ICT-supported integrated care services to promote healthy lifestyles with high potential for comparability and transferability to other sites.

**Trial registration:**

This study protocol has been registered at ClinicalTrials.org (NCT02976064). Registered November 24th, 2016.

**Electronic supplementary material:**

The online version of this article (10.1186/s12913-018-3363-8) contains supplementary material, which is available to authorized users.

## Background

Physical inactivity is acknowledged as a significant public health problem worldwide with kernel implications on major chronic conditions [1, 2]. On the other hand, the positive effects of physical activity (PA) on health outcomes have been extensively demonstrated both in chronic patients [3, 4] and in subjects at risk for developing chronic diseases [5–10].

The growing awareness on the health burden generated by insufficient levels of PA has prompted the interest for deploying community-based initiatives aiming at fostering active healthy living [11]. However, none of the interventions evaluated so far have reached large scale adoption. Moreover, traditional cardiopulmonary rehabilitation interventions temporary enhance aerobic capacity [12–14]; but, they show transient effects, rather low accessibility and no impact on behavioral changes resulting in enhanced PA. In contrast, different small trials seem to suggest efficacy of flexible modular service approaches (i.e. walking, urban training, pedometer-based program, tele-coaching) aiming at promoting active lifestyles [15–19]. Likewise, the analyses of the impact of information and communication technologies (ICT) on healthcare point out their role as enabling tools to support innovative integrated care services through: i) Empowerment of patients for self-management involving adoption of healthy life styles; and, ii) Promotion of efficient interactions among patients, caregivers, community services and healthcare professionals. Thus, ICT-supported services facilitate: i) Patient accessibility; ii) Health coaching; and, iii) Remote follow-up and incentives for patient’s behavioral change [20].

The current protocol relies on the hypothesis that properly tailored self-management services, fully integrated in patient’s health action plan with remote off-line professional support, may induce sustained behavioral changes resulting in active lifestyle behavior. A recent study [21] assessing barriers to deployment of such a collaborative self-management services was useful to identify the requirements for large scale deployment of the novel approach. Accordingly, the current study protocol aims to address those unmet requirements, namely: i) Workflow design of the PA services engaging both patients and healthcare professionals; ii) Enhanced ICT-support; iii) Evaluation strategies including structured indicators during and beyond deployment of the novel services; and, iv) Implementation of innovative business models.

The ultimate aim of the novel PA services for chronic patients and citizens at risk is to generate sustained enhancement of PA with health value generation. Hence, is expected a positive impact on multi-morbidities in terms of: i) Preventing occurrence, ii) Reducing exacerbations, and, iii) Modulating disease progress. The main outcome of the current protocol will be a roadmap for large-scale deployment and assessment of novel collaborative self-management PA services in the region of Catalonia (7.5 million citizens). The approach taken for promotion of PA shall have potential for generalization to other interventions aiming at promoting healthy lifestyles, as part of regional integrated care strategies for chronic patients.

## Methods/design

### Setting

**Fig. 1:**
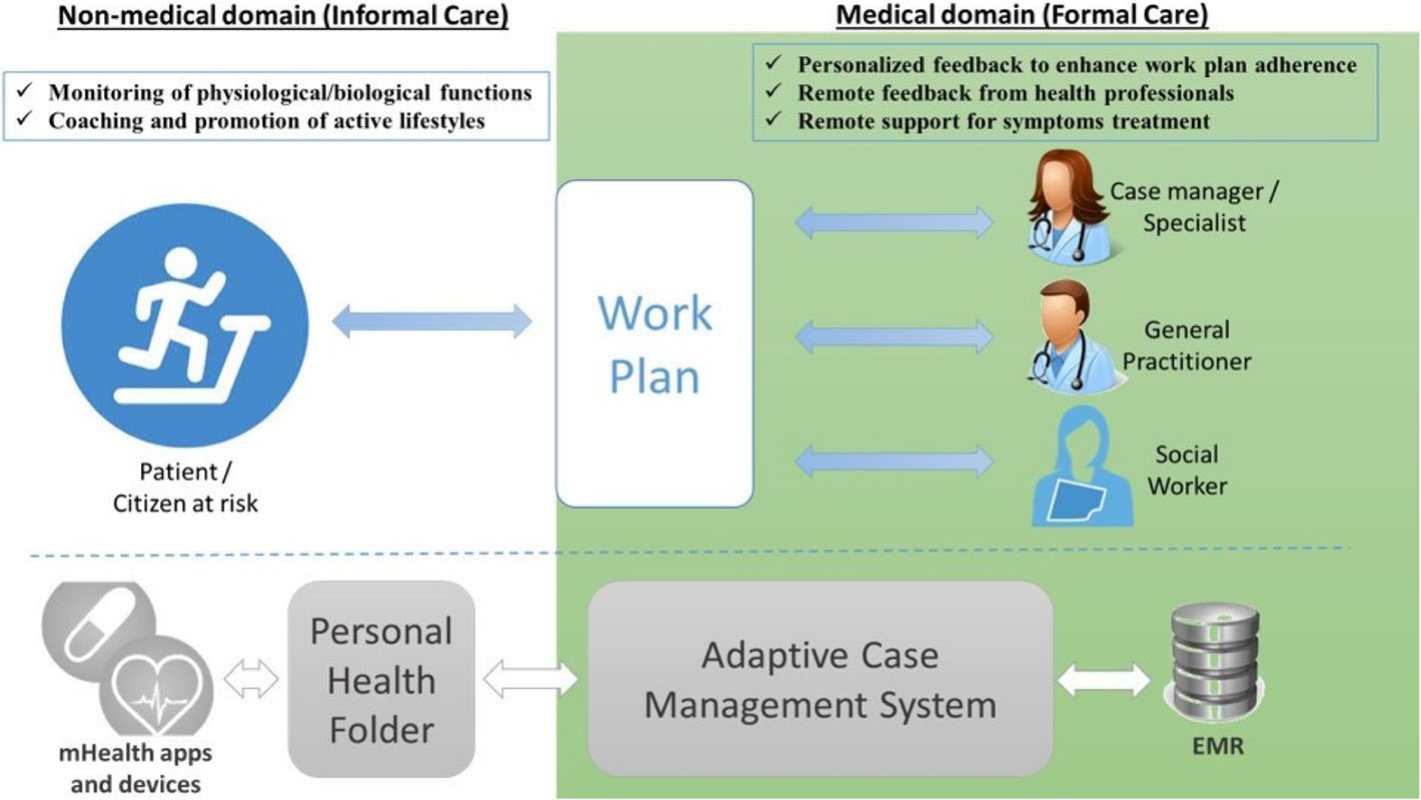
Settings of the implementation studies. The figure shows two interoperable domains with technological elements providing support to the services promoting active lifestyles integrated within the action plan of the patient. On the left, Informal Care area with the patient having access to the Personal Health Folder (PHF) wherein she/he can answer questionnaires, perform monitoring through mHealth apps, and have access to a follow-up reports and tailored educational information, as defined in the work plan (center of the figure). On the right, the Formal Care domain wherein the case manager (physiotherapist) and/or general practitioner has access to an adaptive case management system for work plan prescription, follow-up and coaching. The adaptive case management system supports execution of the patient work plan and provides a bridge of interoperability and collaborative tools among the patient (through the PHF), the case manager and the electronic medical record (EMR)

The protocol has been designed as part of the 2016–2020 Health Plan in Catalonia [22] that involves regional deployment of integrated care services. It has been conceived as a 24 months test bed period plus a third year wherein the initiative will be deployed at regional level. At the end of the second year (2018), three main achievements will be in place. Firstly, the three implementation studies depicted in Fig. 1 will be adopted as mainstream services in one of the four healthcare sectors of the city of Barcelona, Barcelona-Esquerra (AISBE) (540.000 inhabitants). A second milestone will be the elaboration of a plan for generalization of the PA services approach to other non-pharmacological interventions. Thirdly, a roadmap for regional deployment of the PA services in Catalonia (7.5 million habitants) will be launched.

The technological support used in the studies facilitates collaborative work among different healthcare professionals as well as patient empowerment for self-management. This scenario involves deployment of a Digital Health Framework [23, 24] bridging formal and informal care through adoption of a modified version of the current regional personal health folder (PHF) (La Meva Salut® LMS) (https://lamevasalut.gencat.cat/web/guest/pre-login-cps) (Catalonia) as a self-management tool adopted to facilitate patient empowerment for self-care and enhanced monitoring. Moreover, the PHF is linked to the regional shared electronic health record (HC^3^) [25], and provides citizens with an access point to their health information (i.e. discharge reports, imaging, electronic prescription, etc.), medical appointments and potentially for informal health data sources (e.g. mobile health applications, community-based social support services, etc.) as displayed in Fig. 1.

Another key ICT-supporting tool for the current PA protocol is an adaptive case management (ACM) system [26] to support collaborative work among case managers and to facilitate adaptive changes to the continuously evolving needs of the patients within well-structured service workflows. The ACM system will provide support to selection and scheduling of specific tasks throughout the clinical process, as well as facilitation of ad-hoc collaborations with other professionals across healthcare and social support tiers. The ACM platform will be open source and built-up on top of the current health information systems of the different healthcare providers and it will use existing regional interoperability infrastructures alluded to above, such as the shared HC^3^ and the PHF.

Large scale deployment of the current protocol is supported by convergence of different types of resources from: i) Institutional bodies (Department of Health, Generalitat de Catalunya); ii) Healthcare providers (Hospital Clinic de Barcelona); and, iii) EU Grants (see funding section of the manuscript). The *Ethical Committee for Clinical Research* at *Hospital Clínic de Barcelona* approved the study (HCB/2016/0883) which has been registered at ClinicalTrials.org (NCT02976064). Patients’ acceptance and signature of the informed consent will be required to participate in all the studies.

### Aims of the implementation studies

**Fig. 2:**
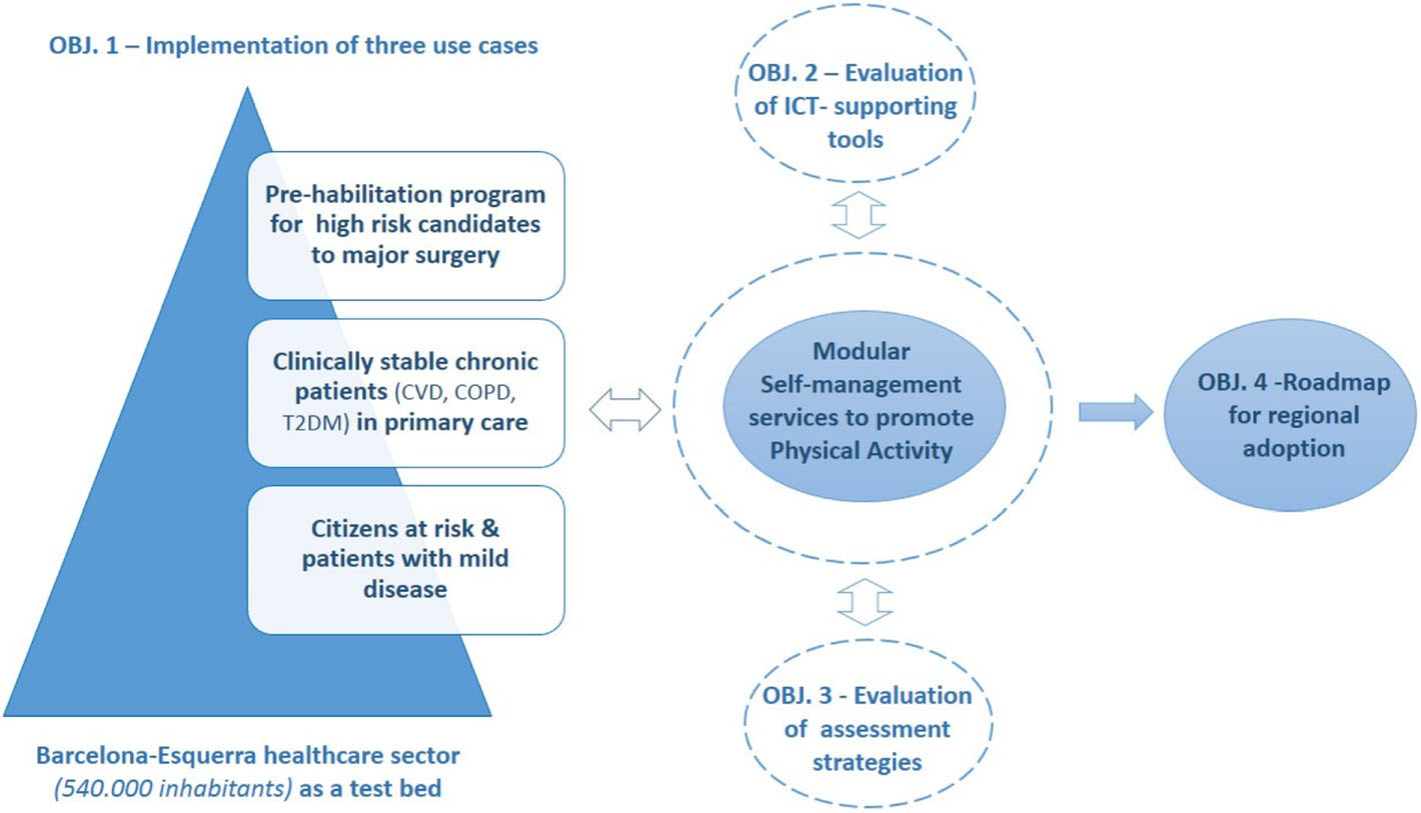
Objectives of the implementation studies. The figure displays the four main objectives of the protocol considered as pivotal steps to achieve regional adoption of collaborative self-management services promoting physical activity across health-care tiers. Deployment of three use cases in one of the healthcare sectors of the city of Barcelona will target chronic obstructive pulmonary disease (COPD), cardiovascular disorders (CVD) and type 2 diabetes mellitus (T2DM). A patient-centered approach will be adopted. ICT stands for information and communication technologies

The protocol addresses the four aims displayed in Fig. 2. Firstly, implementation of three collaborative self-management services designed to promote PA in three study groups representative of different layers of the population-based risk stratification pyramid [27, 28], namely: i) Prehabilitation for high risk candidates to major surgery (Prehabilitation study); ii) Community-based rehabilitation and promotion of PA for clinical stable chronic patients with moderate to severe disease (Rehabilitation study); and, iii) Promotion of PA and healthy lifestyles for citizens at risk and patients with mild disease (Lifestyle study). The second aim is refinement and assessment of the innovative ICT-supporting tools alluded to above. Thirdly, the program adopts the Triple Aim approach [29, 30] for assessment of the three implementation studies and to select key performance indicators (KPI) to be used for follow-up of the services beyond the implementation phase. Finally, the implementation studies will serve to generate a roadmap for regional adoption of the PA services. The sample size will grow according to the capacity of the service. The figures indicated below are realistic estimations.

#### Specific aims of each of the three implementation studies

A recent randomized controlled trial (RCT) carried out in high-risk candidates to major abdominal surgery has demonstrated high efficacy of prehabilitation reducing postoperative complications in these patients [31]. The main aim of the Prehabilitation study (*n* = 1000) in the current protocol is to assess cost-effectiveness of the deployment of the intervention as mainstream service at *Hospital Clinic de Barcelona*.

A previous non-randomized pilot study carried out by the research team [21] identified determinants of adoption of a community-based rehabilitation service for chronic patients, as well as its potential for health-value generation. The main objective of the Rehabilitation study (*n* = 800) is to assess both cost-effectiveness and sustainability of a community-based program combining exercise training and promotion of PA in clinically stable chronic patients showing one or more of the following target disorders: i) Chronic obstructive pulmonary disease (COPD) or other chronic respiratory conditions; ii) Cardiovascular disorders (CVD); and/or iii) Type II diabetes mellitus (T2DM).

Finally, the Lifestyle study (*n* = 1800) will assess large-scale deployment and sustainability of a portfolio of services promoting PA and healthy lifestyles for citizens at risk for the targeted chronic conditions and patients showing mild target disease(s). The study will also assess the impact of the intervention on health-related quality of life.

### Studies design and population

The three implementation studies alluded to above aim at assessing cost-effectiveness and sustainability (business model) of the corresponding intervention as well as to identify factors modulating the success of large scale deployment. To this end, for each implementation study, an intervention group will be compared to a control group (usual care). Briefly, the Prehabilitation study will be deployed at *Hospital Clínic de Barcelona* following a quasi-experimental design using propensity score methods [32] wherein age, gender and population-based health-risk assessment are main matching variables between intervention and control groups. Adjusted Morbidity Groups (GMA) [33, 34] will be used for health risk scoring purposes. The Rehabilitation study will be deployed in the community selecting primary care centers of the healthcare sector as intervention or control units following an open cluster randomized control trial design with a 1:1 ratio. Finally, the Lifestyle study will be a community-based protocol following a quasi-experimental design, as described above.

Inclusion criteria for the Prehabilitation study are: i) Candidates to major elective abdominal, gynecological, cardiovascular, urological and thoracic surgical procedures; ii) Patients presenting high surgical risk defined by: age above 70 years and/or American Society of Anesthesiologists (ASA) score III/IV [35]; and, iii) A tentative surgical schedule allowing for at least 4 weeks for the prehabilitation intervention.

The Rehabilitation study has the following inclusion criteria: i) Patients suffering one or more targeted chronic conditions with moderate-to-severe disease of the main disorder; and, ii) Patients showing history of frequent exacerbations.

Finally, the inclusion criteria for the Lifestyle study are as follows: i) Citizens at risk for chronic conditions; and, ii) Patients showing mild target disease(s). All of them recruited through advertisements and through primary care centers.

Exclusion criteria for the three implementation studies are as follows: i) Emergency surgery (applicable only for the Prehabilitation study); ii) Unstable cardiac of respiratory disease; iii) Locomotor limitation precluding the practice of exercise; and, iv) Cognitive deterioration impeding the adherence to the program.

### Interventions

The entry point to each program, as well as the core management and coordination functions, will be ascribed to the primary care team composed by general practitioner and case manager (physiotherapist). They will be directly in charge of the six common steps of the service workflow, namely: i) inclusion; ii) characterization and re-assessment of patient’s work plan; iii) integration of the intervention into the patient’s action plan; iv) execution of the specific program; v) follow-up/event handling; and, (vi) discharge from the program, as explained in detail in Section 1S.

#### Prehabilitation study

The current protocol aims to rollout the recently reported prehabilitation service [31] to all high-risk candidates to any major surgical procedure.

Candidates for the prehabilitation study fulfilling the inclusion criteria will be identified by the anesthesiologist in the pre-anesthesia visit. All candidates will be assessed in order to address a variety of issues: i) Identify the overall needs of the candidate; ii) Perform a baseline evaluation of his/her fitness and physical activity levels; and, iii) Identify his/her adherence profile, as well as factors and circumstances that may modulate practicalities of the intervention. Personalization of the prehabilitation plan involves the following main actions: i) Calendar and planning of face to face visits and remote (virtual) contacts with healthcare professionals; ii) Intensity/volume of the supervised endurance training program; iii) Threshold of minimum steps per day to promote physical activity; iv) Specific nutritional counseling and intervention (if malnutrition screening universal tool ≥2 [36]); v) Group-oriented psychological interventions (mindfulness); and, vi) Integration of the prehabilitation intervention into the overall work-plan of the patient (see Additional file 1: section “Methods/design”).

#### Rehabilitation study

Health value generation by personalized services including endurance training and promotion of PA will be assessed in chronic patients with target diseases. Briefly, the planned six-month period intervention will have two phases including, as a first step, reassessment of the patient work-plan aiming at optimization of both pharmacological and non-pharmacological therapies. The first phase of the program will start with a motivational interview wherein the intervention will be explained and co-designed with the patient. It will be followed by: i) Supervised endurance training sessions with a flexible duration of 2 to 6 weeks; ii) Promotion of active lifestyle; and, iii) Patient empowerment for self-management of his/her condition aiming at increasing program adherence. The second phase of the intervention will include promotion of PA and self-management using the PHF [37] with remote off-line supervision by a case manager (see Additional file 1: Section 3).

#### Lifestyle study

The main objective is to assess the impact of a program promoting PA and healthy lifestyles in citizens at risk and patients with mild chronic disease. Briefly, key aspects of the intervention are: i) A motivational interview to personalize the intervention; ii) Familiarization of the candidate with the use of the PHF as a self-management tool; and, iii) Assignment of one case manager for off-line remote surveillance of the service. Moreover, the subject will receive information on a portfolio of optional services offered by the service (see Additional file 1: Section “Conclusions”).

### Assessments

**Fig. 3:**
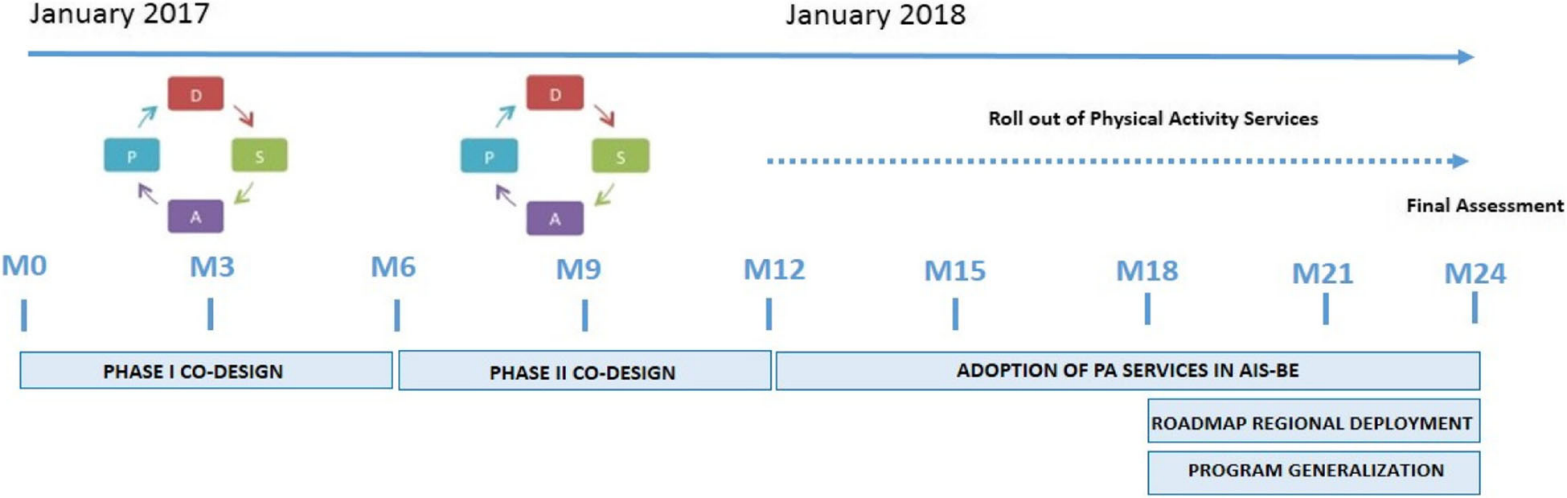
Timeline for program development. The three implementation studies (Fig. 2) will be conducted in parallel. The 24-month lifespan of the programs will be divided in two main phases of approximately one-year duration each. The initial co-design Plan-Do-Study-Act (PDSA) cycle will be devoted to co-design and refinement of the service workflows, set-up and assessment of ICT-supporting tools, and identification of key performance indicators. During the second PDSA cycle, the program will be adopted at pilot level in the healthcare sector. This second phase will be used to fine tune the services, assess and refine ICT-supporting tools, as well as to consolidate the long-term evaluation plans. The two PDSA cycles will have a multidisciplinary approach including patients and professionals with different profiles (i.e. physiotherapists, nurses, general practitioners, medical specialists and technologists). AIS-BE stands for Integrated Care Area of Barcelona-Esquerra (540.000 citizens)

The protocol evaluation will follow a Triple Aim approach [29, 30] considering pre-defined outcome variables for: i) Health and well-being; ii) Experience with care; and, iii) Costs (Table 1). Assessment will be carried out combining empirical questionnaire data collection, information from electronic medical records and registry data. The main study outcome will be twofold: i) Demonstration of cost-effectiveness of the interventions; and, ii) Identification of factors that modulate success of large-scale deployment. Moreover, an ancillary purpose of the implementation studies is to identify and evaluate key performance indicators (KPI) that could be used for long-term follow-up of the service at health system level. To this end, implementation research tools organized within the frame of the Model for ASsesment of Telemedicine applications (MAST) [38] will be considered. During the co-design phases, KPI will be identified and consolidated to be used for long-term assessment of the adoption process (Fig. 3). Most of the variables will be obtained from the automated Catalan Surveillance System [28].

**Table 1:** Main study outcomes of the implementation protocols

Triple Aim	Outcome	Data source & Instrument
Health and well-being	Socio-demographics	Catalan Health Surveillance System & Electronic Medical Records
	Multi-morbidities	Catalan Health Surveillance System & Electronic Medical Records
	Patient Clinical Data	Electronic Medical Records
	Health-related quality of life	SF12 questionnaire
	Healthy lifestyle (Tobacco/Nutrition/Alcohol/Physical Activity)	Electronic Medical Records
	Physical activity	Yale Physical Activity Survey & Monitoring
	Psychological well-being	Hospital Anxiety and Depression scale
	Therapeutic plan (Pharmacological/Others)	Catalan Health Surveillance System & Electronic Medical Records
	Intermediate outcomes (see costs): • Emergency Department visits • General Practitioner visits • Cumulative days per year admitted in hospital • Multiple drugs prescription • Potentially avoidable hospitalizations • Hospital readmissions • Needs for social support	Catalan Health Surveillance System
	Mortality	Catalan Health Surveillance System/Electronic Medical Records
Experience with care	Use of the Personal Health Folder & engagement	Catalan Health Surveillance System
	Home-based technological support	Electronic Medical Records
	Access to integrated care	Catalan Health Surveillance System
	Patient satisfaction & engagement	Electronic Medical Records (non-standard questionnaire)
Costs^a^	Total health and social care cost	Catalan Health Surveillance System
	Primary Care	Catalan Health Surveillance System
	Hospital-related Care • Admissions • Emergency Room consultations • Outpatient specialized care	Catalan Health Surveillance System
	Pharmacy	Catalan Health Surveillance System
	Mental Health	Catalan Health Surveillance System
	Socio-sanitary services	Catalan Health Surveillance System
	Other costs • Respiratory therapies • Dialysis • Rehabilitation • Non-urgent patient transport	Catalan Health Surveillance System

^**a**^ The Catalan Health Surveillance System registries allow allocation of healthcare expenditure to each patient through the Personal Health Identification Number which facilitates analysis of total healthcare expenditure in complex patients

### Regional deployment

The fact that we are building-up on top of a rather mature setting for integrated care adoption reinforces the chances of successful regional deployment of the services. The use of a Plan-Do-Study-Act (PDSA) [34] methodology with iterative cycles shall consolidate refined services at the end of the first year and it will subsequently facilitate regional deployment in Catalonia (Fig. 3). As mentioned, assessment of KPI useful for long-term follow-up of the deployment beyond the implementation studies constitutes a key goal. A roadmap for regional deployment of the three interventions will be produced at the end of the implementation studies.

## Discussion

The present protocol aims to evaluate the determinants of large-scale deployment and adoption of three novel collaborative self-management services promoting healthy lifestyles. We believe that the protocol represents a highly relevant step to foster synergies between non-pharmacological and pharmacological care paving the way for enhanced preventive care for chronic patients.

The study protocol adopts a population-health approach aiming at articulating a test bed that shall facilitate a successful rollout of the three implementation studies at regional level. Synergies across healthcare tiers will be fostered throughout the program deployment. For example, the current hospital-based prehabilitation study will be expanded and enriched with appropriate health-risk assessment tools aiming at enhancing perioperative risk prediction and care at community level. Thus, it should facilitate productive collaborative work between specialized and primary care professionals. Moreover, the program is expected to be highly transferable to other geographical areas.

## Conclusions

The current manuscript reports on the protocol for regional deployment of collaborative self-management services promoting healthy lifestyles that will be carried out in Catalonia during the period 2017–2019.

## Additional file

Additional file 1: Protocol for regional implementation of collaborative self-management services to promote physical activity. This file includes the specific details of each of the three protocols described in the main manuscript. (DOC 531 kb)

## Abbrevations

ACM: Adaptive Case Management
AISBE: Area de Salut Barcelona-Esquerra
ASA: American Society of Anesthesiologists
COPD: Chronic Obstructive Pulmonary Disease
GMA: Adjusted Matching Groups
HC^3^: Historia Clínica Compartida de Catalunya
ICT: Information and Communication Technologies
KPI: Key Performance Indicators
LMS: La Meva Salut
MAST: Model for ASsessment of Telemedicine
PA: Physical Activity
PDSA: Plan-Do-Study-Act
PHF: Personal Health Folder
RCT: Randomized Controlled Trial
