# Supplementary material for: Protocol for regional implementation of collaborative self-management services to promote physical activity
Source: BMC Health Serv Res. 2018 Jul 17;18:560. doi: 10.1186/s12913-018-3363-8 (PMC6050723; doi:10.1186/s12913-018-3363-8)
Supplement: Supplementary file 1 — Protocol for regional implementation of collaborative self-management services to promote physical activity. This file includes the specific details of each of the three protocols described in the main manuscript. (DOC 531 kb) [file 12913_2018_3363_MOESM1_ESM.doc]

**PROTOCOL FOR REGIONAL IMPLEMENTATION OF COLLABORATIVE SELF-MANAGEMENT SERVICES TO PROMOTE PHYSICAL ACTIVITY**

*Anael Barberan-Garcia, et al*.

*On-line supplementary material*

***SECTION 1S.* Commonalities of the three implementation studies**

The entry point to the program, as well as the core management & coordination functions, will be ascribed to the primary care team composed by general practitioner and case manager (physiotherapist and/or nurse). They will be directly in charge of the six common steps of the service workflow *(see Sections 2S to 4S)*: (i) inclusion (step 1); (ii) characterization (step 2) and re-assessment of the patient work plan, as well as (iii) integration of the PA intervention into the program (step 3). The execution of the PA program (step 4) and the follow-up/event handling (step 5) can be shared with wellness centers and SMEs delivering community-based rehabilitation services, among other actors collaborating in the PA program. Discharge from the program (Step 6) and patient allocation into other integrated care services will again be a function of the coordinating team (Primary Care). After completion of a given PA service, the natural pathway will be the transfer of the patient to the program promoting PA addressed to citizens at risk and patients with mild disease.

***SECTION 2S.* Prehabilitation study**

The workflow of the prehabilitation intervention follows six sequential steps described in **Figure 1S** depicted below.


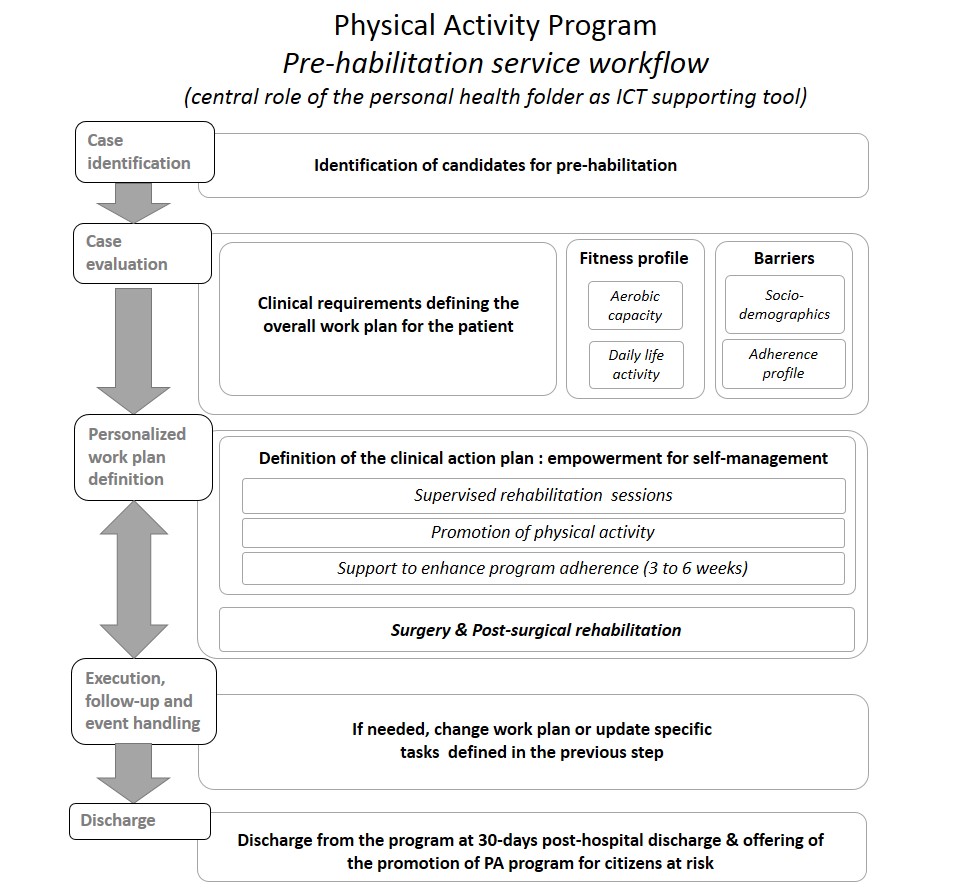


***Figure 1S*** *– Workflow of the Prehabilitation service for high risk candidate to major surgery*

Step 1 – Case identification: Candidates for the pre-habilitation program fulfilling the inclusion criteria will be identified by the anesthesiologist in the pre-anesthesia.

Step 2 – Case evaluation: All candidates will be assessed by the members of the prehabilitation team (anesthesiologist/nurse/physiotherapist) in order to address a variety of issues: i) identify the overall needs of the candidate; ii) perform a baseline evaluation of his/her fitness and physical activity levels; and, iii) identify his/her adherence profile, as well as factors & circumstances that may modulate practicalities of the intervention.

Step 3 – Personalized work plan definition**:** After case evaluation, health professionals will coordinately define the patient’s work plan and its compliance by the ICT-supporting platform. Personalization of the pre-habilitation plan involves the following main actions: (i) Calendar and planning of face to face visits and remote (virtual) contacts with health professionals; (ii) Intensity/volume of the supervised endurance training program; iii) Threshold of minimum of steps per day to promote physical activity; (iv) Specific nutritional counseling and intervention (if MUST ≥ 2)^[5]^; (v) group-oriented psychological interventions (mindfulness); and, (vi) Integration of the prehabilitation intervention into the overall work plan of the patient.

Step 4 – Work plan execution & Step 5 – Follow-up and event handling: Involve all the follow-up tasks, either scheduled or non-scheduled. It includes non-scheduled interactions with the reference professional (usually a physiotherapist) through the personal health folder or the call center. The ICT support to the execution of the workflow facilitating the follow-up of the program and the handling of unexpected events by: (i) fostering patient empowerment for self-management; (ii) enhancing patient adherence to the program; (iii) facilitating remote supervision; and, (iv) allowing patient monitoring.

Moreover, the information gathered through: questionnaires, therapeutic games and wireless monitoring will allow remote (off-line) support by health professionals (physiotherapist, nurse, physician, etc.) using the professional dashboard. In the future, the patient information and communication (ICT) system will be enriched with intelligent apps that will support patient decision making with automated reasoning mechanisms. The later will be designed to comply with legal, safety and ethical constraints.

Postoperative intervention mostly based on non-supervised promotion of physical activity will be planned with a twofold aim: i) to speed-up patient functional recovery; and, ii) decrease rate of hospital-related events.

Step 6 – Discharge: 30 days after hospital discharge, the patient will be discharged from the pre-habilitation service and moved to the PA services for citizens at risk and/or patients with mild disease *(Section 3S)*. Eventually, some patients could be moved to the community-based service addressed to chronic patients with moderate disease.

***SECTION 3S. Community-based enhanced rehabilitation service for patients with moderate to severe target disease(s)***

The workflow of the PA service follows six sequential steps described in **Figure 2S**.


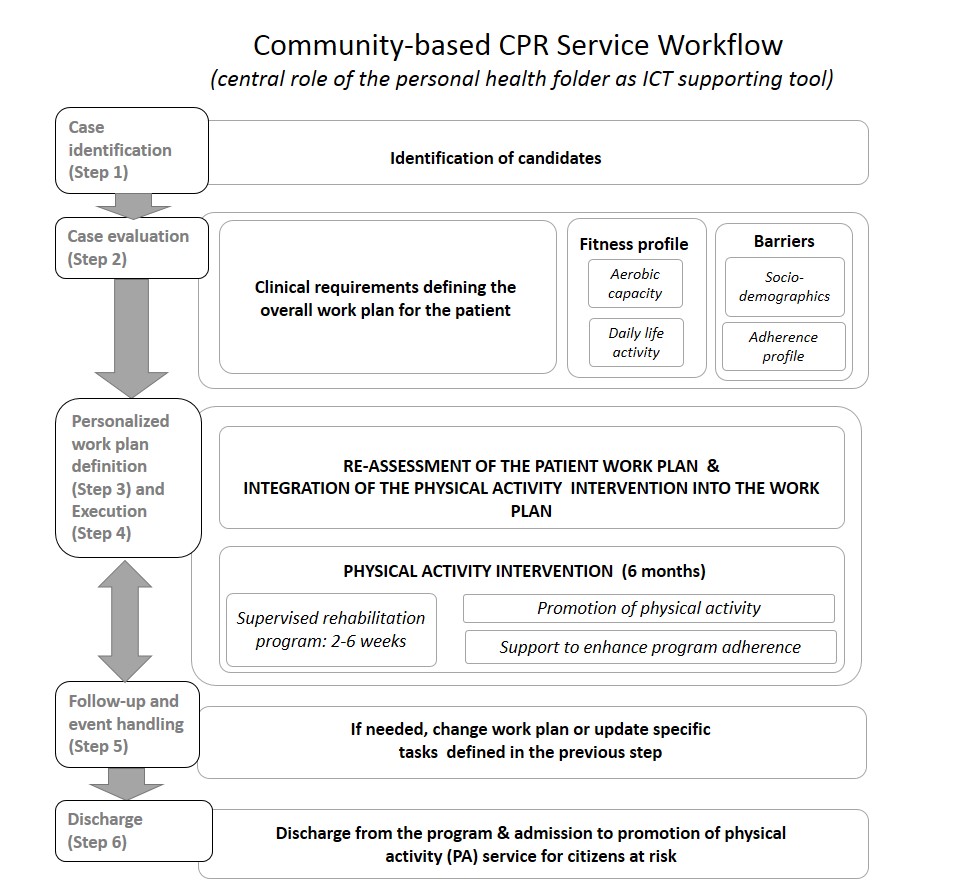


***Figure 2S*** *– Workflow of the PA services addressed to clinically stable chronic patients with target chronic conditions presenting moderate & severe disease (main).*

Step 1-Case identification: The community-based, patient-centered service, will be addressed to cases fulfilling the above mentioned inclusion criteria assessed by the primary care team.

Step 2 -Case evaluation**:** The primary care team (physician/nurse/physiotherapist) will characterize the candidates eligible for the PA service. A holistic characterization of the patient should be performed covering the following areas: (i) Identification of patient requirements defining the work plan; (ii) Assessment of aerobic capacity (6-min walking test, 6MWT) and daily life activities (questionnaire and measurements) in order to personalize type and volume of physical activity; (iii) Identification of factors modulating accessibility (social, logistics, familiar, etc.) and adherence (behavioral profile) to the physical activity program. The aim is to overcome factors that may limit likelihood of success of the program.

Step 3 - Personalized work plan definition: The community-based intervention will include reassessment of the patient’s work plan aiming at optimization of both pharmacological and non-pharmacological therapies through patient empowerment for self-management of his/her health condition. The initial step will consist of an individual motivational interview wherein the PA intervention will be explained and co-designed with the patient. The Physical Activity (PA) intervention will have a total duration of six-months divided in two phases: (i) Phase I (2-6 weeks) will encompass three major types of simultaneous activities: Supervised endurance training sessions, Promotion of active life-style, and Patient empowerment for self-management of his/her condition aiming at increasing program adherence; (ii) Phase II (up to a total of 6 months) will include promotion of PA and self-management using the PHF with remote off-line supervision by a case manager. Promotion of PA will be done using the best environment for each patient (at home and/or at the community environments such as parks, at municipal/private gyms). It will combine two types of activities:

- Community-based activities (individual or group based-sessions) - Walking either in individual or groups sessions (the first objective will be to add 1000 steps to the daily average. After a week, and depending on the daily average steps performed, keep increasing the walking routine until the patient reach 5000 to 6000 steps per day. If the condition of the patient allows it, keep increasing up to 10,000 steps per day).
- Wellness center activities (individual or group-based sessions) - The selection of the exercise routines and activities will depend on the patient’s preferences and clinical profile (this option will be mainly focused in mild patients with physically healthy lifestyle).

Step 4 - Work plan execution & Step 5- Follow-up and event handling: The ICT-support (**Figure 1**) will facilitate the program follow up and the handling of unexpected events, as previously described in Section 1S.

Step 6 – Discharge: At the end of the six-month work plan duration, the patient will be discharged from the program or eventually moved to the PA service addressed to citizens at risk & patients with mild disease(s) described below (Section 4S).

***SECTION 4S.* Promotion of physical activity and healthy lifestyles in citizens at risk and patients with mild disease**

The work-flow of the intervention follows the classical six sequential steps described in **Figure 3S**.


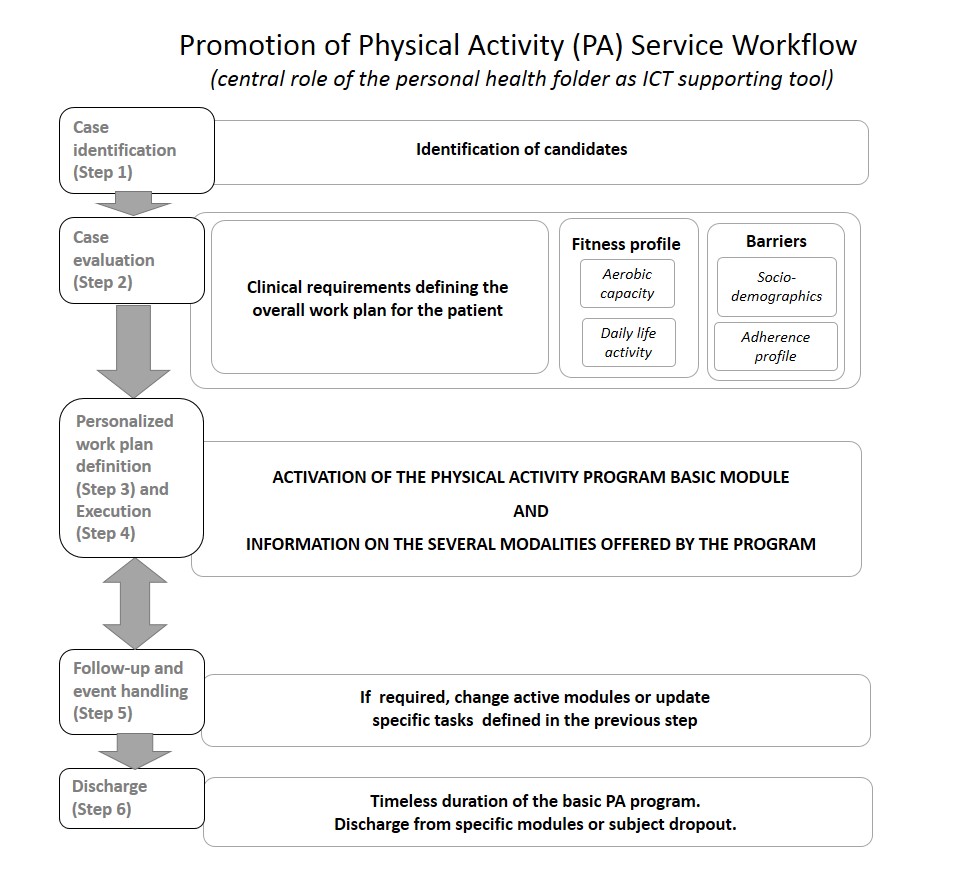


***Figure 3S*** *– Workflow of the PA services promoting physical activity and healthy lifestyles for citizens at risk and chronic patients with mild disease.*

Step 1 – Case identification: Eligible candidates for the PA service are described above in the description of the study group.

Step 2 – Case evaluation: All candidates will be assessed by one of the members of the primary care team (physician/nurse/physiotherapist) in order to cover a variety of issues: i) Identify the overall needs of the candidate; ii) Perform a baseline evaluation of his/her fitness level; and, iii) Identify his/her adherence profile, as well as factors & circumstances that may modulate practicalities of the intervention.

Step 3 – Personalized work plan definition**:** The primary care team will: (i) perform a motivational interview; (ii) familiarize the subject with the use of the personal health folder (PHF) as a self-management tool; and (iii) assign one case manager to perform off-line remote surveillance of the program. Moreover, the subject will receive information on the several optional modalities offered by the program (portfolio of optional services), as well as their associated costs.

Among the optional modalities of this PA service, the following modules are envisaged: i) basic service, as indicated above; ii) attendance to supervised endurance training programs; iii) attendance to group sessions carried out outdoors and/or in wellness centers; and/or, iv) upgraded PA program including sensors and close off-line home-based individual supervision by the case manager. Duration and specificities of the services portfolio will depend on each module.

Step 4 – Work plan execution & Step 5 – Follow-up and event handling: As described in Sections 1S and 2S, the ICT support ill facilitate the program execution, follow-up and the handling of unexpected events.

Step 6 – Discharge: The basic version of the promotion of PA program is conceived for a timeless duration. However, the different modules included in the service portfolio will have specific agendas and associated costs. Moreover, the basic version of the program has been envisaged as the final destination of patients included in the two other complementary programs described in Sections 1S and 2S, respectively: (i) Community-based enhanced rehabilitation (six-month duration) for moderate & severe patients with target disorders; and, (ii) Pre-habilitation program for high risk candidates to major surgical procedures.

**References**

1 Generalitat de Catalunya. Cat@Salut La Meva Salut. Canal personal de salut. at <http://canalsalut.gencat.cat/ca/home_ciutadania/salut_az/c/cat_salut_la_meva_salut/>

2 Cano I, Alonso A, Hernandez C, Burgos F, Barberan-Garcia A, Roldan J, Roca J. An adaptive case management system to support integrated care services: Lessons learned from the NEXES project. J Biomed Inform 2015;55:11-22.

3 Dindo D, Demartines N, Clavien PA. Classification of surgical complications: a new proposal with evaluation in a cohort of 6336 patients and results survey. *Annals of Surgery* 2004;**240**:205–13.

4 2016 Relative value guide book: A guide for anesthesia values. American Society of Anesthesiologists, 2015.

5 Malnutrition Screening Universal Tool. <http://www.bapen.org.uk/pdfs/must/must_full.pdf>
